# Analytical characterization of full, intermediate, and empty AAV capsids

**DOI:** 10.1038/s41434-024-00444-2

**Published:** 2024-02-19

**Authors:** Aisleen McColl-Carboni, Serena Dollive, Sarah Laughlin, Rudenc Lushi, Michael MacArthur, Shanshan Zhou, Jeffrey Gagnon, Christopher A. Smith, Brenda Burnham, Robert Horton, Dimpal Lata, Brianna Uga, Kalyani Natu, Emmanuela Michel, Celia Slater, Evan DaSilva, Robert Bruccoleri, Tim Kelly, James B. McGivney

**Affiliations:** 1 Oxford Biomedica (US) LLC, 1 Patriots Park, Bedford, MA 01730 USA; 2 Congenomics, LLC, Glastonbury, CT USA

**Keywords:** Gene expression analysis, Biophysical methods, Isolation, separation and purification, Gene therapy, Microbiology techniques

## Abstract

Manufacturing of recombinant adeno-associated virus (AAV) vectors produces three types of capsids: full, intermediate, and empty. While there are different opinions about the impact of intermediate and empty capsids on safety and efficacy of AAV products, they are generally considered impurities because they are not the intended fully intact vector product. The presence of these impurities could impact product efficacy due to potential competition with fully packaged AAVs for cellular transduction, as well as have potential implications to patient safety due to increased capsid load during dosing. To determine the impact of intermediate capsids on potency, an AAV preparation was separated into fractions enriched for full, intermediate, or empty capsids. Using a matrix of in vitro (infectivity, gene expression, biological activity) and in vivo potency assays to determine potency as a function of capsid content, our results indicate that while intermediate capsids contribute to the vector genome titer of the product and are equally as infectious as full capsids, they do not contribute to the potency of the AAV product. This study confirms the criticality of reducing and controlling the level of intermediate capsids to ensure a more efficacious AAV product.

## Introduction

AAV-based therapeutics are emerging as novel modalities for the treatment of genetic diseases. The most common method for AAV production involves transient transfection of HEK293 cells, which constitutively express adenovirus E1A and E1B genes, with plasmids carrying the necessary components for assembly of the AAV vector: 1) gene of interest (GOI), flanked by inverted terminal repeats (ITRs), 2) helper genes, which include adenovirus E2A, E2B and E4, and 3) Rep and Cap genes [1, 2].

AAV vectors are structurally complex; the desired AAV vector (i.e., “full” capsid) consists of a protein capsid shell, which is an icosahedral assembly of three viral proteins (VP; VP1, VP2, and VP3) at a molar ratio of approximately 1:1:10, and a DNA payload termed the vector genome (VG) [3]. Along with the intended full capsids, various product-related impurities can be present in AAV vector preparations: empty capsids, which lack the vector genome required for function, and intermediate capsids, which are a heterogeneous mixture of capsids containing sub-genomic vector species, fragments of the plasmids used for transient transfection, or residual host cell DNA (Supplementary Fig. S1) [4–7].

Empty capsids contribute to the overall viral load during clinical dosing [8] and higher viral loads have the potential to exacerbate capsid-triggered immune responses [9]. There is an observed correlation between adverse events and increased viral load in clinical trials for AAV-based therapeutics [10]. Furthermore, therapeutic AAVs are dosed based on VG titer, meaning that while non-functional empty capsids contribute to the overall viral load they do not contribute to clinical efficacy. A common approach to reduce the overall viral load is the targeted removal of empty capsids from the final product which can be achieved using an optimized AAV manufacturing process [11, 12]. Less is currently known about the impact of intermediate capsids on safety and efficacy of AAV products, and their presence in clinical preparations may pose similar risks as described for empty capsids especially if the capsids are identified to be non-functional. This is complicated further by the fact that removal of intermediate capsids remains a fundamental challenge to current purification methods [13].

Due to the challenges outlined above, it is imperative that intermediate and empty capsid impurities are identified, quantified, and controlled in clinical preparations. Central to this are effective analytical methods to measure the levels of these impurities, as well as characterize their potential impact to patients. This report provides a comprehensive biochemical assessment of what is inside intermediate and empty AAV capsids and their impact on in vitro and in vivo potency.

## Materials and methods

The analytical toolbox used for AAV vector profile characterization in this study is summarized in Supplementary Table S1.

### Purified Drug Substance produced at 500 L scale

The vector analyzed in this study is a Clade F hematopoietic stem cell derived AAVHSC-15 [14] containing a single-stranded genome of approximately 3.9 kb long, manufactured by Homology Medicines, Inc (Bedford, MA, USA). Briefly, HEK293 cells were transfected with a triple plasmid construct and purified using a two-column process. The vector material was buffer exchanged and concentrated to yield drug substance. This material was designated AAV-OXBS1.

### Capsid fractionation by preparative ultracentrifugation (prep-UC)

**Fig. 1:**
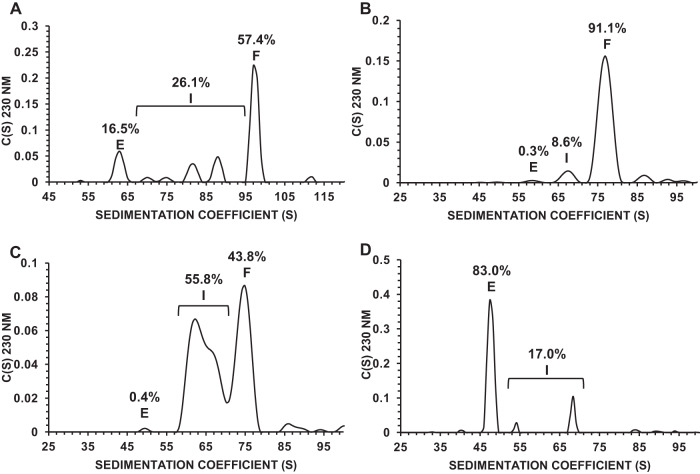
Capsid content determination by AUC. AUC sedimentation distribution plots for rAAV samples pre- and post-preparative ultracentrifugation, *n* = 1 for the assay. **A** OXBS1 AEX product (representative of DS), **B** OXBS1-F pool, **C** OXBS1-I pool, and **D** OXBS1-E pool. The percentage of full capsids (F), intermediate capsids (I) and empty capsids (E) are shown in each figure.

The material was separated into three fractions using prep-UC. Briefly, cesium chloride (JT Baker, Phillipsburg, NJ, USA) was added to an aliquot of AAV-OXBS1 to a final concentration of 3 M. The sample was placed into 10 mL tubes (Beckman Coulter, Indianapolis, IN, USA) and subjected to ultracentrifugation (Optima^TM^ L-90k, Beckman Coulter) for approximately 21 hours at 55,000 rpm and 20 °C. Following ultracentrifugation, the bands corresponding to full (OXBS1-F), intermediate (OXBS1-I) and empty (OXBS1-E) capsids were extracted individually. For enhanced purity, the OXBS1-E and OXBS1-I fractions were subjected to an additional prep-UC step. Each fraction was dialyzed into a phosphate-buffered saline (PBS)-like solution using 10 kDa MWCO gamma-irradiated dialysis cassettes (Thermo Fisher Scientific, Waltham, MA, USA), then concentrated with a 100 kDa MWCO spin concentrator (MilliporeSigma, Burlington, MA, USA). Fractions were then formulated at 3E + 13 capsids/mL in a PBS-like solution. OXBS1-F, OXBS1-I and OXBS1-E samples were then analyzed for capsid content purity by analytical ultracentrifugation (AUC); the resulting component percentages for each sample are shown in Fig. 1.

### Analytical ultracentrifugation (AUC)

Quantification of percent full, intermediate, and empty AAV capsids was performed using an Optima AUC (Beckman Coulter). Analysis of AAV samples by the SEDFIT c(S) model yields a distribution of sedimentation coefficients with each peak in the distribution representing an intact AAV genome or product-related impurity in the form of packaged intermediate VG or residual nucleic acid impurities or empty capsids. Integration of the individual peaks yields the sedimentation coefficient (S) and the relative concentration of each species in the distribution [15]. The linear relationship between S and the length (in nucleotides) of the packaged transgene can be used to predict the S value of a fully packaged AAV.

### Charge detection mass spectrometry (CDMS)

CDMS testing was performed by Megadalton Solutions (Bloomington, IN, USA), according to their procedure (https://megadaltonsolutions.com/) [16]. Briefly, samples were sprayed into a CDMS instrument wherein individual ions were trapped, the charge detected, and the resulting masses were calculated.

### VP purity and ratio

VP purity and VP1:VP2:VP3 ratios were determined by capillary electrophoresis and performed in the presence of sodium dodecyl sulfate (CE-SDS). Samples, reference materials, and standards were heat-denatured in SDS-containing buffer and then separated at 18 kV on a PA800 Plus CE system with an uncoated, fused silica capillary (AB Sciex, Framingham MA, USA). Proteins were detected spectrophotometrically by absorbance at 220 nm.

### Peptide mapping and post-translational modifications (PTMs)

Peptide mapping is a technique for identifying and confirming the primary amino acid sequence of VP proteins and was determined using liquid chromatography mass spectrometry (LC-MS/MS). Test samples were dried down and reconstituted in 8 M guanidine-HCl (Fisher Scientific, Pittsburg, PA, USA). Samples were reduced with a working concentration of 10 mM dithiothreitol (Thermo Fisher Scientific, Waltham, MA, USA) at 25 °C for 30 min and alkylated with a working concentration of 20 mM iodoacetamide (Thermo Fisher Scientific) at 25 °C for 15 min, in the absence of light. Following alkylation, samples were buffer exchanged into 100 mM Tris-Cl, pH 7.5, digested with 2 µg of MS-grade trypsin (Promega, Madison, Wisconsin, USA) at 37 °C for 30 min, then quenched with 1% formic acid. Tryptic-digested peptides (~6 µg) were injected onto an Acquity BEH C18 column (2.1 ×100 mm, 1.7 µm, 300 Å; Waters^TM^ Corporation, Milford, MA) at 60 °C on a Vanquish^TM^ Flex UHPLC (Thermo Fisher Scientific). Peptides were separated using a mobile phase containing 0.1% LCMS-grade formic acid in LCMS-grade water, %A and LCMS-grade acetonitrile, %B (Fisher Scientific). Using a flow rate of 0.4 mL/min and starting ratio of 2% B, a linear gradient was ramped to 5% B from 0 to 2.5 min, 2% B from 2.5 to 5.1 min, 15% B from 5.1 to 20 min, 25% B from 20 to 40 min, 45% B at 40–50 min, 95% from 50 to 55 min, maintained at 95% B for 5 min, decreased to 2% from 60 to 62 min, and maintained at 2% for 70 min to equilibrate.

Data was collected using data-dependent acquisition (DDA, Top 5) in positive mode on a Q Exactive^™^ HF mass spectrometer (Thermo Fisher Scientific). HESI (heated electrospray ionization) source parameters were as follows: in-source CID (collision induced dissociation), 15 eV; sheath gas flow rate, 40; auxiliary flow rate, 20; sweep gas flow rate, 1; spray voltage, 3.80 kV; capillary temperature, 300 °C; S-lens RF, 55.0; and auxiliary gas temperature, 300 °C. Precursor (MS1) scans were obtained using the following parameters: microscans, 5; resolution, 60,000; AGC (automatic gain control) target, 3e6; max IT: 60 ms; scan range, 150–2250 m/z. MS2 scans were obtained using the following parameters: resolution, 15,000; AGC target, 1e5; max. IT, 200 ms; isolation window, 1.2 m/z; normalized collision energy, 27 eV; min. AGC threshold, 2e3; charge exclusion, 1 and >8; dynamic exclusion, 10.0 s.

Data was processed using BioPharma Finder^™^ 3.2 (Thermo Fisher Scientific) and Skyline (MacCoss Lab Software, Version 21.2.0.425). Carbamidomethylation was set as a fixed modification while variable modifications included acetylation (N-terminal and Lys), deamidation and succinimide (Asn and Gln), oxidation (Met and Trp), phosphorylation (Ser, Thr, Tyr), and methylation (Arg). Up to two missed cleavage sites were allowed with a minimum of three consecutive b- or y-ions and a mass error of less than 3 ppm for assignment of peptides and PTMs.

### Vector genome (VG) titer

VG titer was measured by standard droplet digital polymerase chain reaction (ddPCR). All test samples were treated with RNase-free DNase I and proteinase K prior to analysis. Processed samples were amplified in ddPCR^TM^ Supermix (BioRad, Hercules, CA, USA). Primers/probe used were specific for the GOI with binding sites between the middle and 3’ regions of the GOI. Following the droplet generation via an oil:water emulsification, samples were amplified to the endpoint in a thermal cycler and subsequently scanned on a QX200 droplet reader (BioRad). Data were analyzed with QuantaSoft™ software (BioRad).

### Capsid titer

Capsid titer was determined by commercially available AAV9-specific ELISA kit (Progen, Heidelberg, Germany, Catalog No. PRAAV9, https://us.progen.com//AAV9-Titration-ELISA/PRAAV9) and performed according to the manufacturer’s instructions.

### Residual nucleic acids

ddPCR was used to measure residual nucleic acid impurities, including Rep/Cap, manufacturing plasmid sequences and adenovirus E1A. All test samples were treated with proteinase K prior to analysis. Processed samples were amplified in ddPCR Supermix (BioRad) using primers/probe specific for the target sequence. Following the droplet generation via an oil:water emulsification, samples were amplified to the endpoint in a thermal cycler and subsequently scanned on a QX200 droplet reader (BioRad). Data were analyzed with QuantaSoft™ software (BioRad).

### Residual host cell DNA

The amount of residual human DNA was determined by quantitative PCR (qPCR) using a commercially available kit (Thermo Fisher Scientific, Applied Biosystems, Catalog No. A26366, https://www.thermofisher.com/order/catalog/product/A26366?SID=srch-hj-A26366) according to the manufacturer’s instructions. qPCR was performed on the DNA using a primer/probe set directed to a repetitive element in the human DNA and a Quantstudio^TM^ 6 Flex instrument (Thermo Fisher Scientific).

### Nucleic acid sequencing

All three DNA containing samples (OXBS1, OXBS1-F, and OXBS-I) were extracted with the Invitrogen^TM^ PureLink^TM^ Viral RNA/DNA Mini kit with an added DNase step prior to lysis to ensure capture of encapsidated DNA. DNA quality was assessed by TapeStation (Agilent), Nanodrop^TM^ (Thermo Fisher Scientific), and Qubit (Thermo Fisher Scientific). Next generation sequencing was performed on the PacBio Sequel II. SMRTBells were ligated onto DNA and libraries were loaded onto the instrument according to instructions provided by Pacific Biosciences (PacBio; Menlo Park, California, USA).

Data was preliminarily processed with software from PacBio, and bi-stranded CCS calling was performed. Sequencing results were analyzed with custom software. In short, reads were bioinformatically tiled against sequences for the three plasmids used for vector production. Results were enumerated for all the sequence configurations of interest on a presence/absence basis. To further characterize vector genome sequences, a pattern matching scheme was performed to compare all vector genome sequences against common vector genome motifs. Reads were sorted into bins, or buckets, based on observed sequence motif, meaning that similar reads that followed the same motif pattern were group together computationally. Additionally, all vector genome sequences were checked for the presence or absence of the full payload sequence (defined as the sequence expected to be packaged between the ITRs), and the proportion of full payloads were calculated for each sub-bin.

For snapback vector genomes, the sequences around all snapback nucleotides were obtained from the payload section of the reference FASTA file and its reverse complement file, where snapback nucleotide was defined as the 3’ nucleotide on the 5’ subsequence aligning to the payload. The RNAfold program (https://www.tbi.univie.ac.at/RNA; version: 2.5.1) was used to calculate the minimal free energy (MFE) with a sliding window approach for both plus/coding and minus/non-coding strands for all snapback nucleotides in all three samples (OXBS1, OXBS1-F, and OXBS-P). Additional statistical calculations were performed in R statistical software (https://www.r-project.org; version: 4.2.3).

### Infectivity

The infectious titer (TCID_50_) of test samples was performed in HeLa RC32 cells (ATCC, Manassas, VA, USA). Cells were plated in 96-well plates at 3E + 04 cells per well in Dulbecco’s Modified Eagle Medium (DMEM) containing 10% fetal bovine serum and incubated at 37 °C with 5% CO_2_ overnight. At the time of infection, vector aliquots were serially diluted in serum-free DMEM containing adenovirus type 5 (Ad5) at a multiplicity of infection (MOI) of 5. Following a brief incubation at 37 °C, an equal volume of complete medium was added to each well. Cells were lysed 72 h post-infection, and the total cell DNA was extracted and analyzed for VG copies by ddPCR. The primer/probe set used for the VG titer, infectivity and transgene expression assays was identical for all assays.

### In vitro transgene expression

Expressed transgene messenger ribonucleic acid (mRNA) was assessed by two-step reverse transcriptase quantitative polymerase chain reaction (RT‑qPCR). HeLa cells (ATCC) were infected with test samples at a fixed MOI range. Following infection, the RNA was extracted from the cells, DNase-treated and reversed transcribed to complementary DNA (cDNA) by RT-qPCR. A portion of the cDNA was then used in the subsequent qPCR step using specific primers to amplify both the transgene target and an internal control housekeeping target. A delta-delta Ct calculation was performed, and values were plotted against log MOI in SoftMax Pro software (Molecular Devices, San Jose, CA, USA) to obtain a relative gene expression semi-log fit curve. The effective concentration at 50% activity (EC_50_) from the reference was divided by the EC_50_ from the sample and multiplied by 100, yielding the percent relative gene expression (% RGE) result.

### Residual sequence expression

mRNA expression of E1A, E2A, E4, KanR and Rep/Cap was assessed as described in the in vitro transgene expression section above. Ct values for each target were calculated and plotted against MOI in GraphPad Prism Software (version 9; San Diego, CA, USA).

### In vitro potency

The in vitro potency of test samples was determined using a plate-based colorimetric assay to measure enzymatic activity of the expressed protein. HEK293T cells (Takara Bio, San Jose, CA, USA) were co-infected with test samples and Ad5 co-helper at a range of AAV MOIs. Following 48-h incubation at 37 °C with 5% CO_2_, the cells were lysed, and the substrate combined with assay buffer on an assay plate. After overnight incubation of 16–22 h in a dark incubator at 37 °C, stop solution was added and the plate was read at 515 nm on a SpectraMax M5 Microplate Reader (Molecular Devices). The signal generated was directly proportional to the amount of enzymatic activity and was plotted against the MOI in SoftMax software and fit to a four-parameter logistic (4-PL) model. The sample curves were constrained to the reference and the final percent relative potency (%RP) results were determined as the reference EC_50_ divided by sample EC_50_ and multiplied by 100.

### In vivo transgene expression

A colony of B6.129(Cg) wild type mice was maintained in the animal facility at Homology Medicines under standard laboratory conditions, and animals were supplied with food and water ad libitum. This study consisted of four cohorts each with four 4- to 6-week-old male mice: formulation buffer alone, OXBS1-F (9E + 13 capsid/kg), OXBS1-I (9E + 13 capsid/kg), and OXBS1-E (9E + 13 capsid/kg). About five weeks after initial dosing, the mice were sacrificed, and the target organs were harvested and stored at -80 °C. Total RNA was isolated from the tissues using the RNeasy Kit (QIAGEN, Germantown, MD, USA) and 1 ng of RNA was used for each sample as input for reverse transcription into cDNA. GOI copies per reaction were calculated following ddPCR and normalized to nanogram (ng) per reaction to generate a final value of GOI copies per ng RNA for each mouse. The final % RGE result was determined as the average GOI copies per ng RNA for the sample group (OXBS1-I or OXBS1-E) divided by the average GOI copies per ng RNA for the OXBS1-F group and multiplied by 100.

## Results

### Using prep-UC to fractionate full, intermediate, and empty AAV capsids into enriched capsid populations

A single-stranded rAAV vector lot containing a heterogenous mixture of full (57.4%), intermediate (26.1%), and empty (16.5%) capsids (Fig. 1a) was subjected to prep-UC using a cesium chloride gradient to generate bands for each capsid population (Supplementary Fig. S2). Two rounds of separation were performed and the bands corresponding to full (OXBS1-F), intermediate (OXBS1-I), and empty (OXBS1-E) capsids were extracted individually. It should be noted that because both the OXBS1-I and OXBS1-F bands were so close together, to ensure the entire OXBS1-I band was extracted, it was inevitable that a subpopulation of OXBS1-F would also be present.

The first step in analytical characterization was to quantify capsid content purity for each extracted sample. Several accepted analytical methods can be used to determine the packaging profile of rAAV capsids, including using the ratio of the VG titer compared to the capsid titer, transmission electron microscopy, AUC, and CDMS [17]. AUC was chosen since this method provides sufficient resolution to distinguish between full, intermediate, and empty capsids and, therefore, allow quantitation of each species [15]. Figure 1 shows AUC size distribution profiles reflecting the relative size and abundance of the separated species in each sample. The OXBS1-F fraction was enriched for full capsids (91.1%) with a low level of intermediate capsids (8.6%) (Fig. 1b). The OXBS1-I fraction was a mixture of intermediate (55.8%) and full (43.8%) capsids (Fig. 1c). The OXBS1-E fraction was enriched for empty capsids (83.0%) with a low level of intermediate capsids (17.0%) (Fig. 1d).

### CDMS as an orthogonal method for quantification of full, intermediate and empty AAV capsids and evaluation of capsid charge state

**Fig. 2:**
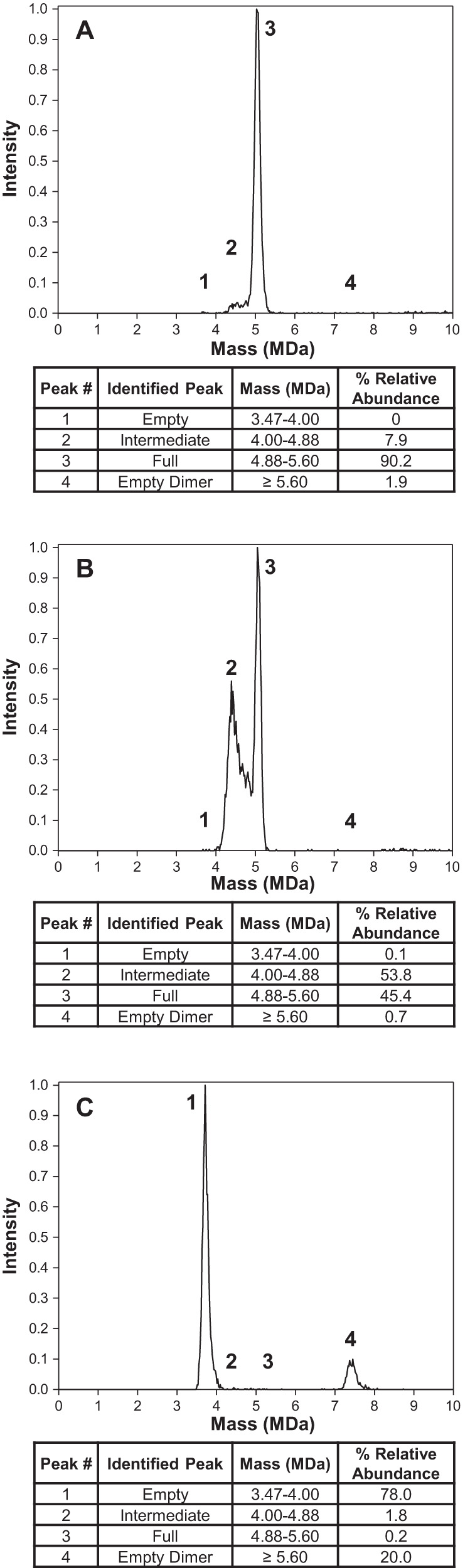
Capsid content determination by CDMS. CDMS mass distribution plots for rAAV samples post-preparative ultracentrifugation. Tables below the figure detail the identified peak, mass range (MDa) and % relative abundance, *n* = 1 for the assay. **A** OXBS1-F, **B** OXBS1-I, and **C** OXBS1-E.

CDMS analyzes AAV capsid content through the differences in charge and mass between empty and full capsids. Figure 2 shows CDMS mass size distribution profiles reflecting the relative mass and abundance of the separated species in each sample. The genome mass (1.34 MDa) was calculated by subtracting the mass of empty capsid peak (3.71 MDa) from the full capsid peak (5.05 MDa) and aligned closely to the expected genome mass for this GOI (1.28 MDa). Narrow peak widths for OXBS1-F (Fig. 2a) and OXBS1-E (Fig. 2c) suggested homogenous packaging of full and empty capsids, respectively. In contrast, the peak width for intermediate capsids in OXBS1-I (Fig. 2b) was much wider (4.00–4.88 MDa) and suggested more heterogenous packaging. The genome mass of the major peak in OXBS1-I, calculated by subtracting the mass of intermediate capsid peak (4.39 MDa) from full capsid peak (5.07 MDa), was roughly half the genome length at 0.68 MDa. The OXBS1-E fraction consisted primarily of empty capsids at 3.71 MDa. However, a second peak of 20% relative abundance was identified with 7.41 MDa mass, which correlates with an empty capsid dimer. Interestingly, this peak was quantified as intermediate capsids by AUC, which highlights the importance of having orthogonal analytical methods to characterize quality attributes. The relative abundance of full, intermediate, and empty capsids in OXBS1-F (Fig. 2a), OXBS1-I (Fig. 2b) and OXBS1-E (Fig. 2c) fractions calculated by CDMS were highly comparable to that determined for AUC as shown in Fig. 1.

CDMS can also determine charge state distribution and provide orthogonal information about the capsid structure. Despite the substantial mass increase from empty capsids (3.71 MDa) to intermediate capsids (4.39 MDa) to full capsids (5.05 MDa), the average charge increased only slightly (around 10 units) (Supplementary Table S2). This phenomenon was observed previously [18] and is thought to be consistent with a small expansion of the capsid with larger genome size and with all the genomes being fully internalized. The largest difference in charge was observed for empty dimer (7.41 MDa; 293.4 units) compared to empty monomer (3.71 MDa; 158.6 units) in the OXBS1-E fraction with the likelihood of charge (for the empty dimer) nearly doubling due to a doubling in overall size.

### Determination of VP purity and VP1:VP2:VP3 ratio by CE-SDS

**Fig. 3:**
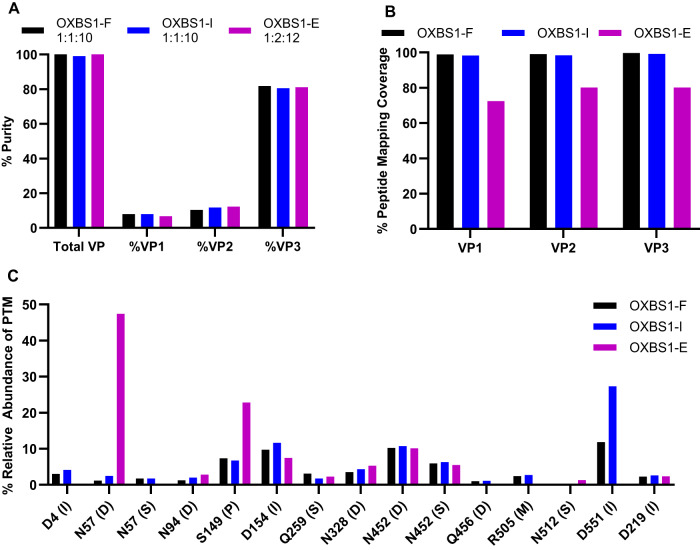
Characterization of VP purity, VP1:VP2:VP3 ratio, and post-translational modifications of full, intermediate, and empty capsids by CE-SDS and LC-MS/MS. **A** VP purity and VP ratio of OXBS1-F, OXBS1-I, and OXBS1-E were determined by CE-SDS. **B**, **C** Peptide mapping of the VP proteins present in OXBS1-F, OXBS1-I, and OXBS1-E pools was determined by LC-MS/MS. **B** Sequence coverage was confirmed according to the primary amino acid sequence using BioPharma Finder™ software (Thermo Fisher Scientific) and **C** post-translational modifications were identified using both BioPharma Finder™ and Skyline. *N* = 1 for the assay. VP Viral Protein, I aspartic acid isomerization, D deamidation, S succinimide, P phosphorylation, M methylation.

The AAV capsid is composed of three viral proteins (VP1, VP2, and VP3) assembled into an icosahedron at a molar ratio of approximately 1:1:10 of VP1:VP2:VP3 [3]. The relative expression levels of VP1, VP2 and VP3, as well as PTMs of the amino acid side chains, can be affected by the production method used and deviation from the 1:1:10 ratio may result in alterations in the amount of PTMs, potentially impacting AAV in vivo activity [19, 20]. To determine whether VP heterogeneity is observed at the capsid level, OXBS1-F, OXBS1-I and OXBS1-E samples were characterized by CE-SDS. OXBS1-F and OXBS1-I samples were comparable in VP purity (100%) and VP1:VP2:VP3 ratio (1:1:10) (Fig. 3a). However, the OXBS1-E samples had a slightly lower amount of VP1 and a slightly higher amount of VP2, resulting in a 1:2:12 ratio.

### Identification and quantification of PTMs in full, intermediate, and empty AAV capsids by LC-MS/MS

**Fig. 4:**
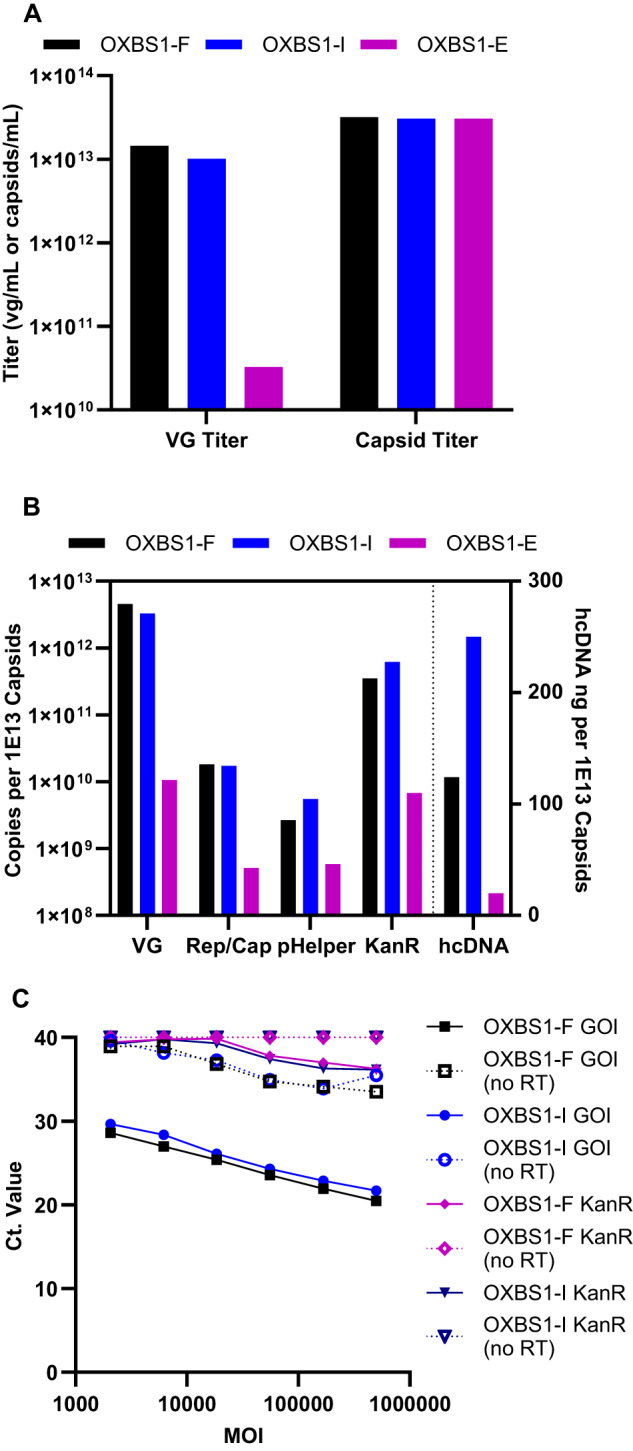
Measurement of VG titer, capsid titer, process-related impurities, and residual sequence expression in HeLa cells. **A** VG titer and capsid titer for OXBS1-F, OXBS1-I, and OXBS1-E pools were determined by ddPCR and ELISA, respectively. **B** Levels of plasmid-derived DNA impurities (Rep/Cap, Helper plasmid, KanR; left y-axis) and host cell DNA (right y-axis) were determined by ddPCR and qPCR, respectively. Results for each target were normalized to 1E + 13 capsids to allow direct comparison between samples. **C** mRNA expression of GOI and KanR was measured across a dose range of MOIs for OXBS1-F and OXBS1-I in HeLa cells by RT-qPCR. Each point represents the average of *n* = 2 wells. A no RT control (dotted lines) was run for each to confirm the assay is specifically measuring gene expression.

VP integrity and PTMs of the enriched capsid samples were characterized using LC-MS/MS. The determined amino acid sequence matched the expected amino acid sequence for AAVHSC-15 with ≥98% coverage of VP1, VP2 and VP3 for OXBS1-F and OXBS1-I samples (Fig. 3b). The amino acid sequence coverage for VP1, VP2 and VP3 for OXBS1-E was 72–80%. Capsid titers were similar for all three enriched samples (3E + 13 capsids/mL) (Fig. 4a).

The percent relative abundance for PTMs is shown in Fig. 3c; identified modifications include isoaspartic acid isomerization, asparagine/glutamine succinimide, asparagine/glutamine deamidation, serine phosphorylation and arginine methylation. The abundance of most AAV VP PTMs were comparable across the OXBS1-F, OXBS1-I, and OXBS1-E samples. The most common spontaneous PTM, asparagine deamidation, can result in an aspartic acid or isoaspartic acid modification. Deamidation was observed at comparably low levels across full, intermediate, and empty capsid populations, except for VP1 residue N57, which was 47% deamidated in the OXBS1-E fraction compared to 1% and 2% N57 deamidation for OXBS1-F and OXBS1-I, respectively. In addition, phosphorylation at S149 was approximately 3-fold higher for OXBS1-E (23%), when compared to OXBS1-F (7%) and OXBS1-I (7%) fractions.

Taken together, the data suggests that full and intermediate AAV capsids have a highly similar profile of PTMs. Empty capsids, on the other hand, had higher deamidation and phosphorylation, specifically at N57 and S149, respectively. The presence of S149 phosphorylation, which is present on both the VP1 and VP2 regions, may play a role in cell signaling and/or trafficking while N57, unique to VP1, is located on a domain that has been shown to play a role in AAV in vivo and in vitro transduction [20]. The aforementioned PTMs are less abundant in intermediate and full capsids and may influence the packaging profile – or lack thereof – for empty capsids. These characteristics provide insight into the biophysical properties of both full and intermediate capsids and provide further insight into why separation and purification of these species remains a current challenge.

### Determination of titer for vector genome and residual impurities

Following purification, a portion of AAV capsids containing fragments of nucleic acids other than the fully intended VG may be present as product-related impurities (Supplementary Fig. S1). These impurities, which can include fragments of the VG, host cell DNA and production plasmid DNA, have the potential to impact the efficacy and safety of the product. A comprehensive characterization of these impurities was performed to assess the potential risks of product-related impurities to patients.

The VG titer of the non-fractionated AAV-OXBS1 sample and the enriched fractions (OXBS1-F, OXBS1-I, and OXBS1-E) was determined by ddPCR. The VG titers of OXBS1-F, OXBS1-I and OXBS1-E samples were 1.45E + 13, 1.01E + 13 and 3.26E + 10, respectively (Fig. 4a). All samples were formulated at 3E + 13 capsids/mL. The VG titer for OXBS1-I was ~70% less than OXBS1-F. When considering the percent full capsids by AUC for OXBS1-I (43.8%) and OXBS1-F (91.1%), the percent full for OXBS1-I was 48% less than OXBS1-F. Overall, these findings suggest that the intermediate genomes in the OXBS1-I sample may also be contributing to VG titer.

The amounts of total residual nucleic acid process-related impurities are shown in Fig. 4b. Sample results were normalized to capsid titer to generate residuals per 1E + 13 capsids. The plasmid-derived DNA impurities (Rep/Cap, Helper plasmid and KanR) were present at comparable levels in the OXBS1-F and OXBS1-I pools at 2–4 logs lower than vector genome. Adenovirus E1A levels were below the limit of quantitation (<1E + 07 copies/mL). Residual host cell DNA impurities are fragments of human genomic DNA unintentionally packaged within AAV capsids [4]. In a previous study, AAV vectors produced by transient transfection contained up to 0.30% host cell DNA [21]. Our data shows that residual host cell DNA is enriched in the OXBS1-I fraction, indicating that the host cell fragments are smaller than the optimal 4.7 kB packaging size. All DNA impurities were low in the OXBS1-E empty capsid population (<7E + 09 copies/1E + 13 capsids).

### Evaluation of residual impurity sequence expression in vitro

To determine whether the encapsidated DNA impurities were capable of being expressed upon transduction into cells, a residual gene expression assay was performed following transduction of HeLa cells with a dose response curve of OXBS1-F and OXBS1-I samples. Dose-dependent expression of the GOI was observed across the fixed MOI range for OXBS1-F and OXBS1-I (Fig. 4c). No signal was detected for E1A, E2A, E4 or Rep/Cap residual gene expression in both OXBS1-F and OXBS1-I samples (qPCR Ct value was undetermined; data not shown). Dose-dependent expression of KanR mRNA was detected in the reverse transcriptase (RT) group but not the in the absence of RT (no RT group), indicating that this was specifically detecting KanR mRNA expression (Fig. 4d). However, KanR mRNA expression was only observed below baseline 40 Ct level at very high VG/cell (above 5.6E + 04) and up to 15 Ct higher when compared to expression of the GOI. It is uncertain whether such expression would be triggered in vivo with much lower expected VG/cell, although further studies are warranted to confirm this.

### Determining the proportion of VG and impurities by next generation sequencing (NGS)

DNA impurity analysis above shows the heterogenous nature of DNA packaging for this AAV preparation. To characterize encapsidated DNA further, DNA was extracted from OXBS1, OXBS1-F, and OXBS1-I samples and prepared for long-read next generation sequencing using the PacBio platform. Read size distributions corresponded with AUC fractionation (Supplementary Fig. S3). Sequencing reads were compared to the packaging plasmids and sorted in bulk into bins based on sequence identity: VG (Full or intermediate); Reverse Packaging; ITR only; Plasmid Backbone; Plasmid Backbone and VG; RepCap; pHelper; and Chimera (which contained reads that had subsequences from multiple plasmids) (Table 1). When compared to unfractionated OXBS1, the OXBS1-F fraction was enriched for VG sequences, whereas the OXBS1-I fraction was enriched for non-VG sequences. As was observed for the ddPCR data above, absolute read counts for RepCap and pHelper containing reads were 2–4 orders of magnitude lower than the vector genome containing reads in all three samples.

**Table 1:** Summary of bulk NGS sequence attribution.

Classification	OXBS1	OXBS1-F	OXBS1-I	
VG	80.20%	89.93%	72.28%	
ReversePackaging	9.82%	5.86%	12.79%	
ITR Only	0.39%	0.13%	0.52%	
Plasmid Backbone	0.06%	0.02%	0.08%	
Plasmid Backbone and VG	5.26%	2.41%	8.74%	
RepCap	1.51%	0.73%	1.40%	
pHelper	0.08%	0.01%	0.06%	
Chimera	2.68%	0.91%	4.12%	
Sequences Analyzed	100.00%	100.00%	100.00%	

Total read counts of detected species were normalized to generate percentages of VG and residual species and normalized to total classified reads. Less than 2.1% of total reads were unable to be classified for each sample.

Reads classified, or determined to be, as VG in Table 1 were further analyzed to determine the proportion of full, snapback VG [22], and truncated VG subpopulations in each sample (Table 2). When compared to unfractionated OXBS1, the OXBS1-F fraction was enriched for full VG, whereas the OXBS1-I sample was enriched for snapback partial VGs (Table 2). Truncated partial VGs appeared at similar rates across all three samples. Unexpectedly, most of the classified intermediate VG species captured were snapback partials, not truncated partials. Both truncated VGs and snapbacks contain partial vector genome sequences and are typically unable to produce a full recombinant product in the case of recombinant gene therapies; therefore, both types of subgenomes can be considered contaminants [7]. However, unlike truncated VGs, snapbacks have a hairpin-like genome and contain ITRs on both the 5’ and 3’ genome ends, which are important for episome formation [23, 24]. Further studies are needed to explore the nuclear durability of both truncated and snapbacks VGs and assess if high proportions of snapbacks within the intermediate genome subpopulation is a general phenomenon.

**Table 2:** Summary of vector sequence attribution for vector reads by NGS sequencing.

	OXBS1		OXBS1-F		OXBS1-I	
	% of classified reads	% of bin that is full	% of classified reads	% of bin that is full	% of classified reads	% of bin that is full
Full VG	63.03%	93.76%	81.86%	94.58%	43.37%	91.24%
Snapback Partial VG	9.63%	0.03%	2.90%	0.14%	18.24%	0.05%
Truncated VG	1.40%	0.58%	1.45%	0.38%	1.36%	0.24%
Other	6.53%	15.20%	3.85%	20.71%	9.83%	4.60%
Total	80.59%	74.59%	90.07%	86.86%	72.80%	55.00%

Reads classified as VG were further classified by pattern matching to determine the proportion of Full, Snapback, and Truncated subpopulations. Any reads that did not match any predefined pattern were sorted into the “Other” category. All read percentages are normalized to the total number of classifiable reads (left column), including non-vector sequences. The proportion of reads in each category that fully aligned to the payload portion of the VG reference (defined as the complete sequence between the ITRs) was then calculated (right column).

To characterize the snapback genomes further, the 5’ payload aligning segment was mapped for each read to determine the nucleotide at which alignment to the full expected VG ended. The positions where these alignments ended for all snapback reads were enumerated individually for the plus and minus strands (Supplementary Fig. S4). Surprisingly, the location of the terminal nucleotide was extremely discrete. In the OXBS1-I sample, three nucleotides in the reference accounted for over 25% of the snapback nucleotides in + strand reads, suggesting that there is a specific phenomenon responsible for formation of the snapback structure. Local folding structure was calculated across the vector genome with a sliding window approach. Despite observing significant predicted structure in some snapback nucleotides, several high-count snapback nucleotides did not have a high degree of predicted local folding structure (Supplementary Fig. S4). While the occurrence of snapback structure appears to be influenced by local folding structure, it does not explain the existence of all major breakpoints. Both C and G nucleotides were significantly enriched at breakpoints on the plus and minus points for all vectors (X^2^ *p*-value < 2.2E−16). In aggregate, these data indicate that there is a characteristic process to snapback genome formation [7]. However, a more diverse panel of AAV vector genomes would be needed to clarify the mechanism.

### Evaluating the impact of intermediate capsids on in vitro and in vivo potency

AAV potency consists of three parts: 1) transduction of the AAV capsid into target cells; 2) expression of the transgene within the cell; and 3) biological activity of the expressed protein. A full matrix of in vitro potency assays (infectivity, transgene expression and biological activity) was developed for this rAAV product to evaluate the impact of intermediate and empty capsids on potency. The infectivity assay determines the infectious titer of the sample and is reported relative to the VG titer (i.e., VG/IU). The in vitro transgene expression and biological activity assays determine the percent relative transgene expression (%RGE) and percent relative potency (%RP), respectively, when compared to a product-specific reference standard.

**Fig. 5:**
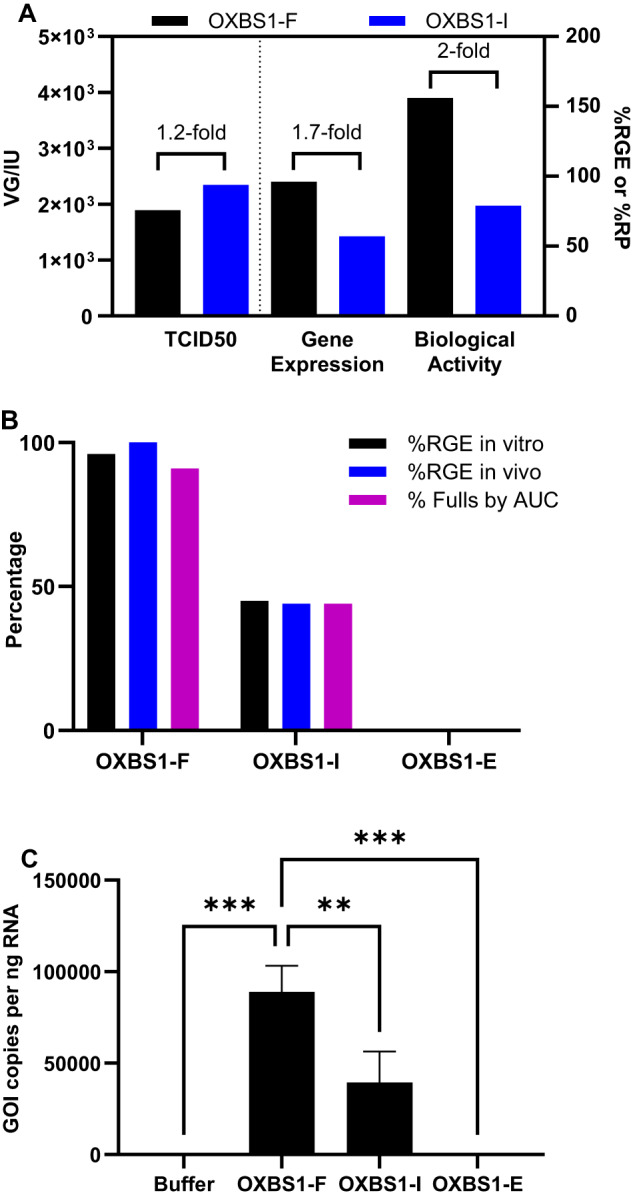
Evaluating the impact of intermediate capsids on in vitro and in vivo potency. **A** Infectivity (VG/IU; left y-axis), transgene expression (%RGE; right y-axis) and biological activity (%RP; right y-axis)= were determined for OXBS1-F (black bars) and OXBS1-I (blue bars) samples using the nominal VG titer to determine potency as a function of vector genome. *N* = 1 for each sample. **B** Transgene expression was determined in vitro (%RGE; black bars) and in vivo (%RGE; blue bars) for OXBS1-F, OXBS1-I, and OXBS1-E samples using the nominal capsid titer to determine potency as a function of capsid content. % Full capsids were determined by AUC (purple bars). *N* = 1 for all assays. **C** Transgene expression was determined in liver tissue 5 weeks-post infusion of mice with 9E13 capsids/kg of OXBS1-F, OXBS1-I, or OXBS1-E samples. Formulation buffer was used as a vehicle control. GOI copies per ng RNA was determined by ddPCR. One-way analysis of variance (ANOVA) was used to determine any statistically significant differences between the means of the capsid groups. Error bars are SEM. *N* = 4 mice per cohort.

In vitro potency results for OXBS1-F and OXBS1-I samples are shown in Fig. 5a. The sample VG titer was used for each assay to allow determination of potency as a function of vector genome. Infectivity results (VG/IU) were comparable for OXBS1-F and OXBS1-I samples and within the expected variability for this method, indicating that the samples enriched for full or intermediate capsids were equally infectious. However, sample divergence was observed for transgene expression (%RGE) and biological activity (%RP) where the OXBS1-I fraction was 1.7-fold and 2-fold less potent than the OXBS1-F sample, respectively. The fact that transgene expression and biological activity was even observed in the OXBS1-I sample was likely due to the presence of 43.8% full capsids. To test this theory, OXBS1-F, OXBS1-I and OXBS1-E samples were next tested in the transgene expression assay by running all samples using the OXBS1-F vg titer (1.45E + 13 vg/mL). Since all three samples had an identical capsid titer of 3E + 13 capsids/mL, this resulted in equal loading of capsids per cell and allowed for determination of transgene expression as a function of capsid content. As shown in Fig. 5b, the %RGE was highest for the OXBS1-F sample (96%) and correlated well with % full capsids by AUC (91.1%). The OXBS1-I sample was 45% RGE, which correlated well with AUC results for % full in this sample (43.8%). The OXBS1-E sample, which had no full capsids, was 0% RGE.

Potency as a function of capsid content was also determined in vivo. Mice were infused with an equivalent dose of 9E + 13 capsid/kg of either OXBS1-F, OXBS1-I or OXBS1-E. This dose was expected to result in robust transgene expression in the liver. VG transcript levels in liver samples were determined in a ddPCR transgene expression assay using the same primer/probe set used for VG titer, infectivity, and in vitro transgene expression assays. The GOI copies per ng RNA were calculated and plotted for each group in Fig. 5c. When dosed at equivalent capsids/kg, the OXBS1-F sample was significantly more potent than the OXBS1-I or OXBS1-E samples. This is not entirely surprising since the OXBS1-F cohort had the highest vg/kg of all the groups (OXBS1-F, 4.1E + 13 vg/kg; OXBS1-I, 3.0E + 13 vg/kg; OXBS1-E, 9.6E + 10 vg/kg). As shown in Fig. 5b, the %RGE for OXBS1-F (100%), OXBS1-I (44%) and OXBS1-E (0%) samples correlated well with % full capsids by AUC (91.1%, 43.8% and 0%, respectively).

These results together suggest that while intermediate AAV capsids (containing mostly snapback partial VGs) are capable of infecting cells, they are not transcribed into mRNA to generate a functional protein. It is therefore imperative when analyzing the function of partial VG sequences to leverage a full matrix of assays that evaluate every step of the potency lifecycle i.e., transduction, expression, and biological activity.

### Comparability of AUC, CDMS, NGS and potency results

We next compared the analytical results obtained for the AUC, CDMS, PacBio sequencing, and potency methods. For this analysis, we took the PacBio total read counts of full-sized VG (i.e., ITR through ITR), partial-sized VG (i.e., snapback, truncated or sequences categorized as other that did not contain a full payload) or non-VG (i.e., Rep/Cap, pHelper, Plasmid Backbone, Reverse Packaging, Chimeric sequences, etc.) and normalized each to the total classifiable reads to generate a % full VG, % partial VG and % non-VG value, respectively, by NGS (Supplementary Table S3). A % intermediate genomes were calculated by combining % partial VG and % non-VG groups.

The % full VG results determined by NGS for each sample were remarkably similar to the % full capsids measured by AUC and CDMS, as well as the %RGE measured by in vitro and in vivo transgene expression methods (Table 3), strongly supporting that the full peak quantified by AUC and CDMS contains predominately functional full-length ITR-through-ITR vector. The most striking comparison was observed for the OXBS1-I sample, where the % full (44% by AUC, 45% by CDMS, 44% by NGS; Table 3) and % intermediate (56% by AUC, 54% by CDMS, 56% by NGS; Figs. 1,  2, and Supplementary Table S3) were identical between methods. NGS sequencing was able to determine that the intermediate peak of the OXBS1-I fraction is comprised of both intermediate VGs (predominately snapback partials) and non-VG sequences. Thus, together, AUC, CDMS and NGS analysis provide a comprehensive understanding of the vector profile of the product. Furthermore, these results strongly support that the potency observed in the OXBS1-I sample is attributable to the presence of full VGs in this sample and that the intermediate VGs themselves are not functional.

**Table 3:** Comparison of results for AUC (% full capsids), NGS (% full VG), in vitro transgene expression (%RGE) and in vivo transgene expression (%RGE).

	OXBS1-F	OXBS1-I	OXBS1-E
% Full Capsids (AUC)	91	44	0
% Full Capsids (CDMS)	90	45	0
% Full VG (NGS)	83	44	ND
%RGE in vitro	96	45	0
%RGE in vivo	100	44	0

Results for each assay are *n* = 1. Full NGS VGs were defined as either sequences that contained a full VG matching pattern or a more complex pattern that contained a fully aligning vector payload.

## Discussion

The field of AAV gene therapy is rapidly evolving, with multiple products already approved and several more progressing through clinical development. With this comes the necessity to more fully understand the AAV characteristics that can impact product quality, clinical safety, and efficacy. During the manufacturing of rAAV vectors, intermediate and empty capsid product-related impurities are co-produced alongside full capsids. While there are different opinions about the clinical impact of intermediate and empty capsids [25–29], they are generally considered impurities because they are not the intended fully intact vector product. Significant progress in the industry has been made to reduce empty capsids through optimization of the AAV purification process. However, intermediate capsids are technically challenging to separate from full capsids, as was our experience in this study. It is, therefore, imperative that AAV products are evaluated for the presence of intermediate capsids and, if present, that they are extensively characterized.

In this study, samples were enriched for full, intermediate, or empty capsids and subjected to extensive analytical characterization. Characterization at the capsid level suggests that intermediate and full AAV capsids have remarkably similar chemical properties, including VP1:VP2:VP3 ratio, PTMs, and average capsid charge, which may explain why these capsids co-purify during downstream processing.

Characterization at the genomic level showed that intermediate capsids for this product contained predominately snapback VGs with a low level of truncated partial VGs. In theory, the VG titer for intermediate VG sequences could be undercalculated if the selection site for primer probes is missing on a truncated genome and influence the VG to infectious titer ratio (VG/IU). To mitigate this, the same primer probe set was used for VG titer and VG/IU to provide a generalized comparison of the full and intermediate infectivity. These intermediate VG sequences contributed to the VG titer of the product and were equally as infectious as full VG-containing capsids but were not functional in our in vitro and in vivo potency assays. Furthermore, we were able to demonstrate a direct link between % full capsids observed by AUC and CDMS, % full VG by NGS, and % relative gene expression, both in vitro and in vivo, and to determine that the intermediate VGs were not functional for this single stranded construct.

In conclusion, this study highlights the importance of fully characterizing AAV vectors across capsid, genome, and functional levels to ensure the best quality vector is delivered to patients. Key to this analysis is robust analytical methods that can accurately quantify each critical quality attribute. Using a combination of AUC, CDMS, NGS, and in vitro and in vivo potency methods, our data suggests that while the intermediate VGs for the construct used in this study were not functional, they contributed to the overall VG titer of the product; an observation that has direct implications when it comes to overall viral load during dosing and ultimately patient safety. This study confirms the criticality of achieving a high percent full vector to generating an AAV product that is safe and efficacious. Improved vector quality can be accomplished through optimization of vector design and bioreactor processes to reduce the biosynthetic generation of intermediate AAV capsids [2] as well as improvement of the purification process to reduce empty capsids and ensure a high-quality vector is delivered to patients.

### Supplementary information

Supplemental Material

## Data Availability

Data generated and analyzed during this study can be found within the published article and supplementary files, and additional data are available from the corresponding author upon reasonable request.

## References

[1] Bulcha JT, Wang Y, Ma H, Tai PWL, Gao G (2021). Viral vector platforms within the gene therapy landscape. Signal Transduct Target Ther.

[2] Van Lieshout LP, Rubin M, Costa-Grant K, Ota S, Golebiowski D, Panico T (2023). A novel dual-plasmid platform provides scalable transfection yielding improved productivity and packaging across multiple AAV serotypes and genomes. Mol Therapy Methods Clin Dev..

[3] Samulski RJ, Muzyczka N (2014). AAV-mediated gene therapy for research and therapeutic purposes. Ann Rev Virol.

[4] Wright JF (2014). Product-related impurities in clinical-grade recombinant AAV vectors: characterization and risk assessment. Biomedicines.

[5] Schnödt M, Büning H (2017). Improving the quality of adeno-associated viral vector preparations: the challenge of product-related impurities. Hum Gene Ther Methods.

[6] Penaud-Budloo M, François A, Clément N, Ayuso E (2018). Pharmacology of recombinant adeno-associated virus production. Mol Ther Methods Clin Dev.

[7] Zhang J, Guo P, Yu X, Frabutt DA, Lam AK, Mulcrone PL (2022). Subgenomic particles in rAAV vectors result from DNA lesion/break and non-homologous end joining of vector genomes. Mol Ther Nucleic Acids.

[8] Food and Drug Administration (FDA) Cellular, Tissue, and Gene Therapies Advisory Committee (CTGTAC) Meeting #70: Toxicity Risks of Adeno-associated Virus (AAV) Vectors for Gene Therapy (GT), September 2-3. https://www.fda.gov/media/151599/download 2021.

[9] Ertl HCJ (2022). Immunogenicity and toxicity of AAV gene therapy. Front Immunol.

[10] Kishimoto TK, Samulski RJ (2022). Addressing high dose AAV toxicity - ‘one and done’ or ‘slower and lower’?. Expert Opin Biol Ther.

[11] Gimpel AL, Katsikis G, Sha S, Maloney AJ, Hong MS, Nguyen TNT (2021). Analytical methods for process and product characterization of recombinant adeno-associated virus-based gene therapies. Mol Ther Methods Clin Dev.

[12] Frenkel R, Tribby D, Boumajny B, Larson N, Sampson M, Barney C (2022). ACUVRA: Anion-exchange chromatography UV-ratio analysis - A QC-friendly method for monitoring adeno-associated virus empty capsid content to support process development and GMP release testing. AAPS J.

[13] Joshi PRH, Bernier A, Moço PD, Schrag J, Chahal PS, Kamen A (2021). Development of a scalable and robust AEX method for enriched rAAV preparations in genome-containing VCs of serotypes 5, 6, 8, and 9. Mol Ther Methods Clin Dev.

[14] Smith LJ, Ul-Hasan T, Carvaines SK, Van Vliet K, Yang E, Wong KK Jr, et al. Gene transfer properties and structural modeling of human stem cell-derived AAV. Mol Ther. 2014;22:1625–34. 10.1038/mt.2014.107 PMC4435483 24925207

[15] Burnham B, Nass S, Kong E, Mattingly M, Woodcock D, Song A (2015). Analytical ultracentrifugation as an approach to characterize recombinant adeno-associated viral vectors. Hum Gene Ther Methods.

[16] Barnes LF, Draper BE, Chen YT, Powers TW, Jarold MF (2021). Quantitative analysis of genome packaging in recombinant AAV vectors by charge detection mass spectrometry. Mol Ther Methods Clin Dev.

[17] Werle AK, Powers TW, Zobel JF, Wappelhorst CN, Jarrold MF, Lyktey NA (2021). Comparison of analytical techniques to quantitate the capsid content of adeno-associated viral vectors. Mol Ther Methods Clin Dev.

[18] Barnes LF, Draper BE, Jarrold MF (2022). Analysis of thermally driven structural changes, genome release, disassembly, and aggregation of recombinant AAV by CDMS. Mol Ther Methods Clin Dev.

[19] Rumachik NG, Malaker SA, Poweleit N, Maynard LH, Adams CM, Leib RD (2020). Methods matter: standard production platforms for recombinant AAV produce chemically and functionally distinct vectors. Mol Ther Methods Clin Dev.

[20] Frederick A, Sullivan J, Liu L, Adamowicz M, Lukason M, Raymer J (2020). Engineered capsids for efficient gene delivery to the retina and cornea. Hum Gene Ther.

[21] Lecomte E, Tournaire B, Cogné B, Dupont JB, Lindenbaum P, Martin-Fontaine M (2015). Advanced characterization of DNA molecules in rAAV vector preparations by single-stranded virus next-generation sequencing. Mol Ther Nucleic Acids.

[22] Xie J, Mao Q, Tai PWL, He R, Ai J, Su Q (2017). Short DNA hairpins compromise recombinant adeno-associated virus genome homogeneity. Mol Ther.

[23] Duan D, Sharma P, Yang J, Yue Y, Dudus L, Zhang Y (1998). Circular intermediates of recombinant adeno-associated virus have defined structural characteristics responsible for long-term episomal persistence in muscle tissue. J Virol.

[24] Yan Z, Zak R, Zhang Y, Engelhardt JF (2005). Inverted terminal repeat sequences are important for intermolecular recombination and circularization of adeno-associated virus genomes. J Virol.

[25] Wright JF (2014). AAV empty capsids: for better or for worse?. Mol Ther.

[26] Fuentes C, Staudhammer J, Wright JF, Paulk N, and Cross S. Beyond empty and full: Understanding heterogeneity in rAAV products and impurities. Dark Horse Consulting Group white paper; 2022.

[27] Gao K, Li M, Zhong L, Su Q, Li J, Li S (2014). Empty virions in AAV8 vector preparations reduce transduction efficiency and may cause total viral particle dose-limiting side-effects. Mol Ther Methods Clin Dev.

[28] Flotte TR, Gao G (2017). AAV is now a medicine: We had better get this right. Hum Gene Ther.

[29] Sihn CR, Handyside B, Liu S, Zhang L, Murphy R, Yates B (2021). Molecular analysis of AAV5-hFVIII-SQ vector-genome-processing kinetics in transduced mouse and nonhuman primate livers. Mol Ther Methods Clin Dev.

