# Supplementary material for: Analytical characterization of full, intermediate, and empty AAV capsids
Source: Gene Ther. 2024 Feb 19;31(5-6):285–94. doi: 10.1038/s41434-024-00444-2 (PMC11090809; doi:10.1038/s41434-024-00444-2)
Supplement: Supplementary file 1 — Supplemental Material [file 41434_2024_444_MOESM1_ESM.docx]

**Supplementary Fig. S1**


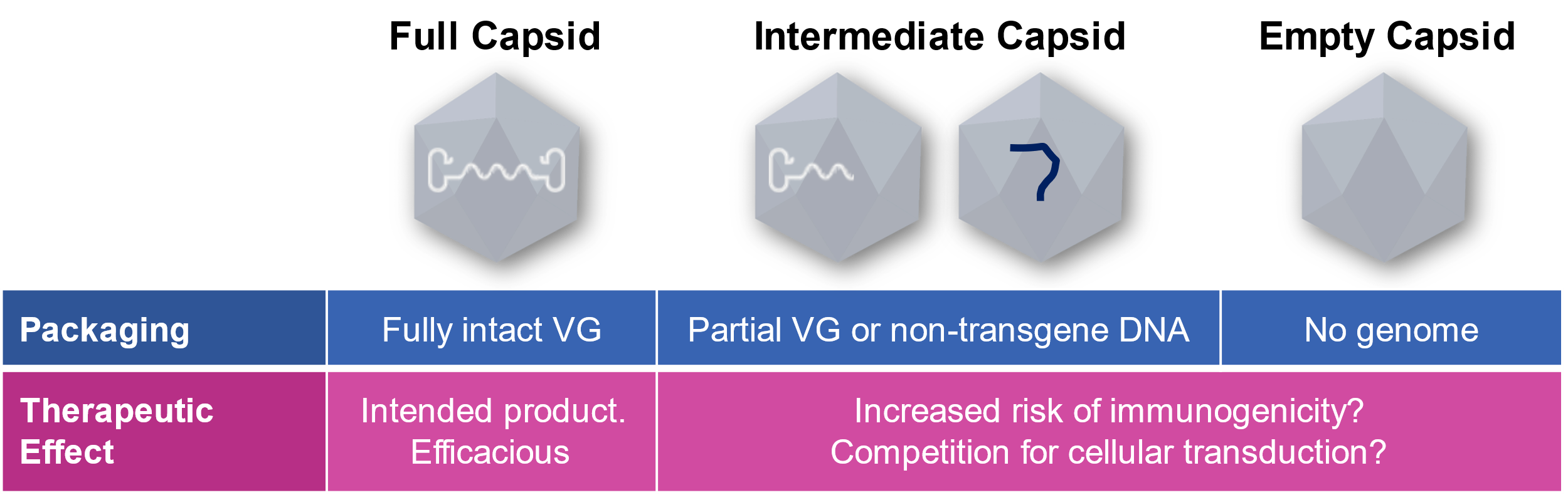


**Supplementary Fig. S1: Overview of AAV capsids generated during rAAV production.** In addition to full capsids which contain the intended fully intact VG, AAV capsids that contain partial VG or non-transgene DNA (i.e., intermediate AAVs) or contain no genome (i.e., empty capsids) can be present as product-related impurities.

**Supplementary Fig. S2**


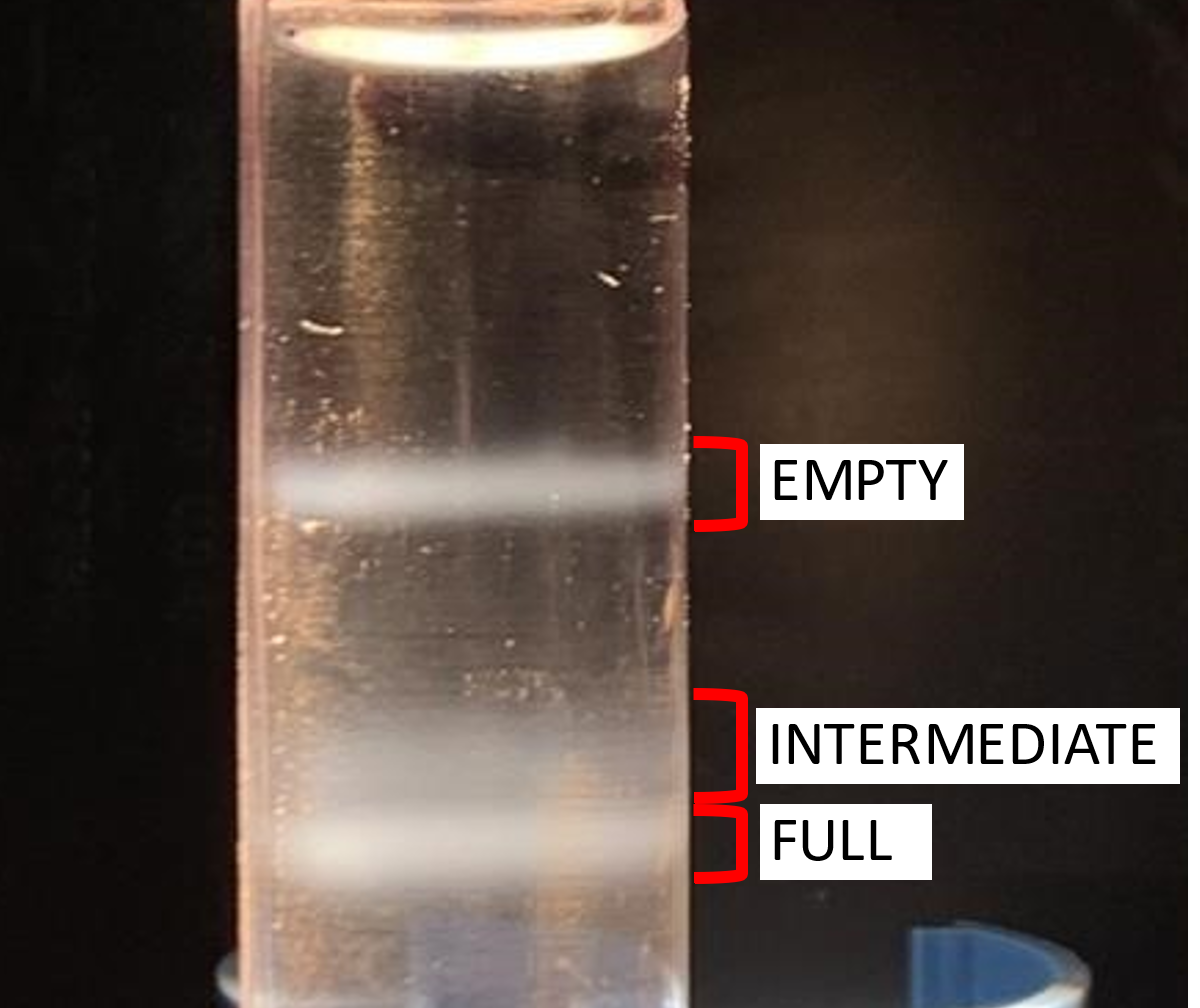


**Supplementary Fig. S2: AAV Capsid Fractionation by Preparative Ultracentrifugation.** An AAV vector lot containing a heterogeneous mixture of full, intermediate, and empty capsids was separated into distinct bands by preparative ultracentrifugation using a cesium chloride gradient. The bands corresponding to full (OXBS1-F), intermediate (OXBS1-I) and empty (OXBS1-E) capsids are labeled and were extracted individually prior to analytical characterization.

**Supplementary Fig. S3**


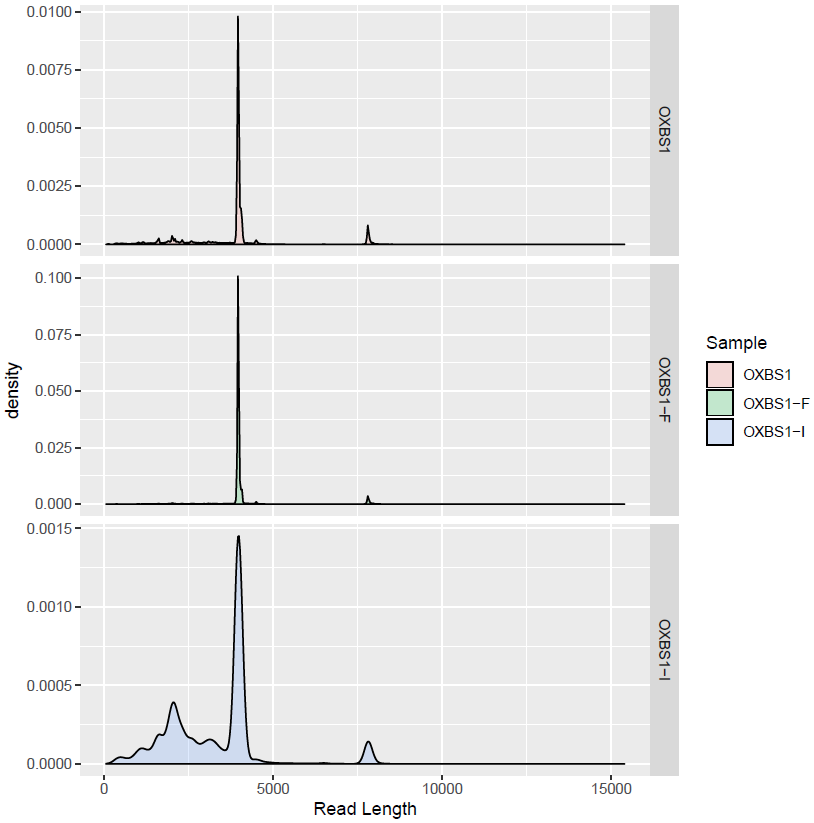


**Supplementary Fig. S3: Read Size Distribution.** Kernal densities of the lengths of all ccs reads successfully called by SMRTLink.

**Supplementary Fig. S4**

**A.**
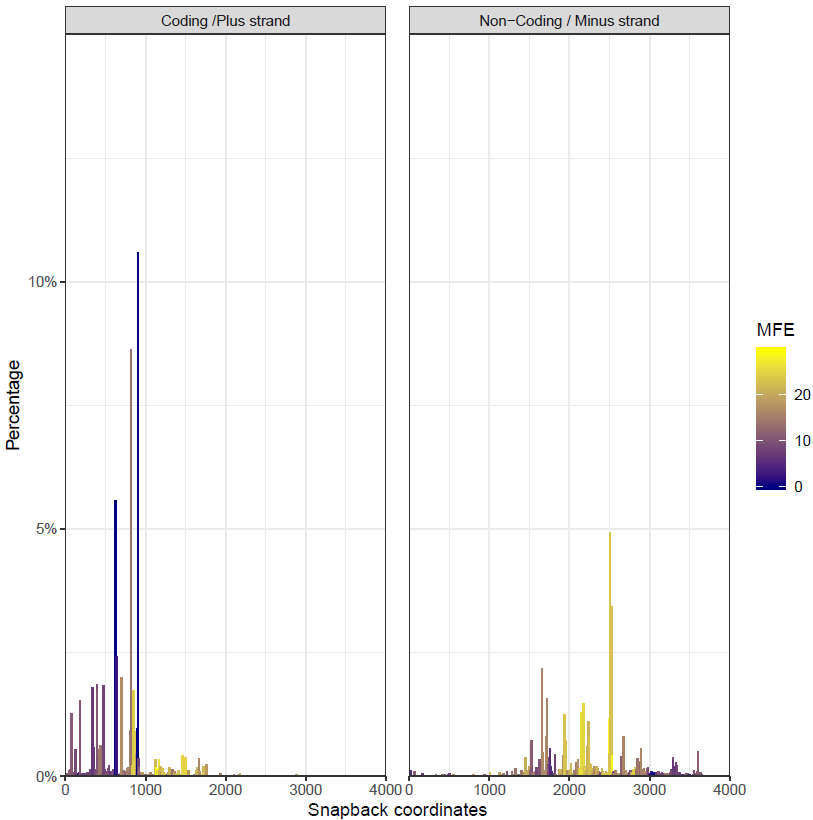


**B.**
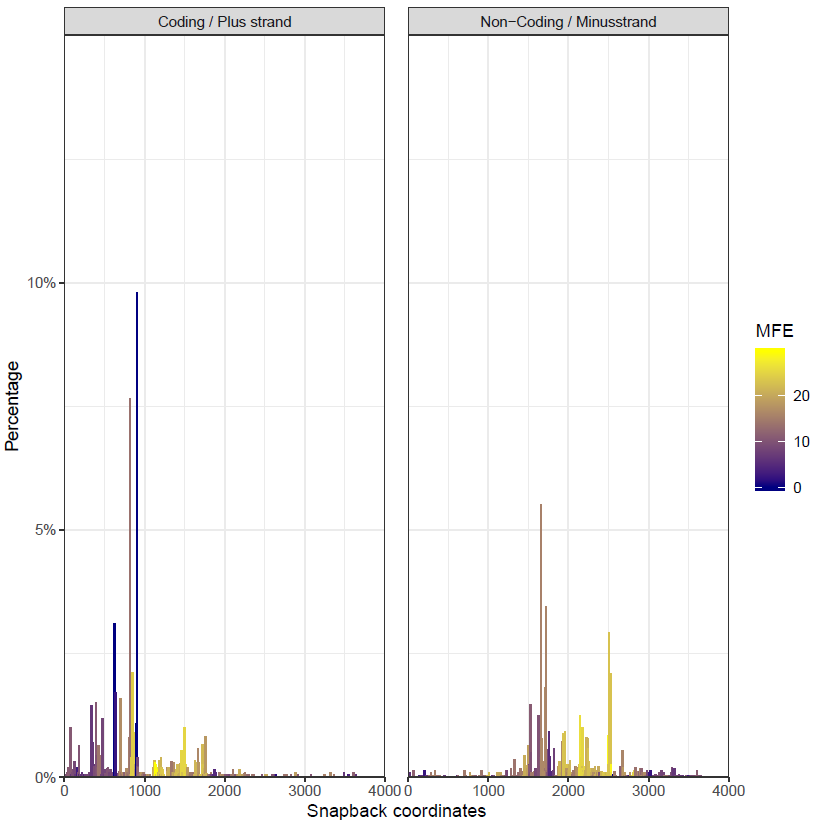


**C.**
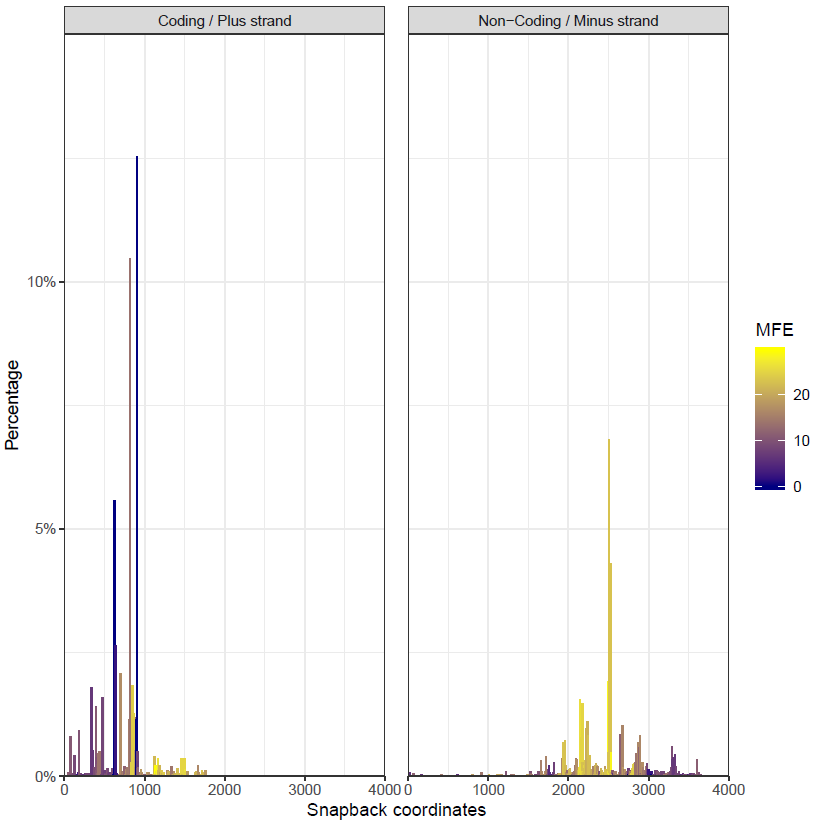


**Supplementary Fig. S4: Histograms of snapback nucleotides for + and - strands.** A. OXBS1. B. OXBS1-F. C. OXBS-I. Snapback nucleotides are defined as the 3’ most aligning nucleotide to the 5’ segment of a snapback vector genome. Calculations were performed by custom scripts in R and Python. Bars are colored by local DNA secondary structure as MFE score calculated by RNAfold. Higher MFE scores indicate a higher probability of local folding structure.

**Supplementary Table S1**

**Supplementary Table S1. Analytical Toolbox for Full Vector Profile Characterization of AAV Products.**

| Attribute Class | Quality Attribute | Method |
| --- | --- | --- |
| **Content** | VG titer | ddPCR |
|  | Capsid titer | ELISA |
| **Strength** | Infectivity | TCID50 (in vitro) |
| **Potency** | Transgene expression | mRNA by RT-qPCR (in vitro) |
|  | Biological Activity / Functional Potency | Product-specific (in vitro and/or in vivo) |
| **Purity** | VP purity & VP1:VP2:VP3 ratio | CE-SDS |
|  | Percent empty, intermediate and full capsids | AUC and CDMS |
|  | Residual host cell DNA | qPCR |
|  | Residual helper plasmid DNA | ddPCR |
|  | Residual Rep/Cap | ddPCR |
|  | Residual E1A | ddPCR |
|  | Residual KanR | ddPCR |
|  | Residual gene expression | mRNA by RT-qPCR (in vitro) |
| **Identity** | Capsid identity | LC-MS/MS (peptide mapping for post-translational modifications) |
|  | Sequence identity | NGS Sequencing |

**Supplementary Table S2**

**Supplementary Table S2. Determination of Average Capsid Charge by CDMS.**

|  |  |  | **Average Charge** | | |
| --- | --- | --- | --- | --- | --- |
| **Peak #** | **Identified Peak** | **Mass (MDa)** | **OXBS1-F** | **OXBS1-I** | **OXBS1-E** |
| 1 | Empty | 3.47-4.00 | 158.2 | 165.3 | 158.6 |
| 2 | Intermediate | 4.00-4.88 | 166.4 | 166.4 | 169.2 |
| 3 | Full | 4.88-5.60 | 167.9 | 168.0 | 178.1 |
| 4 | Empty Dimer | > 5.60 | 288.9 | 278.2 | 293.4 |

**Supplementary Table S3**

**Supplementary Table S3. Calculation of % Full VG and % Intermediate Genomes by NGS Sequencing.** The total read counts of full-sized VG (i.e., ITR through ITR), partial-sized VG (i.e., snapback, truncated or sequences categorized as other that did not contain a full payload) or non-VG (i.e., Rep/Cap, pHelper, Plasmid Backbone, Reverse Packaging, Chimeric sequences, etc.) were normalized to the total classifiable reads to generate a % Full VG, % Partial VG and % Non-VG value, respectively, by NGS. % Intermediate genomes were calculated by combining % Partial VG and % Non-VG groups.

|  | **OXBS1** | **OXBS1-F** | **OXBS1-I** |  |
| --- | --- | --- | --- | --- |
| Full VG | 64.03% | 82.66% | 43.83% |  |
| Partial VG  (Snapback + Truncated) | 16.56% | 7.40% | 28.97% |  |
|  |  |  |  |  |
| Non-VG | 19.41% | 9.94% | 27.21% |  |
| Intermediate Genomes  (Partial VG + Non-VG) | 35.97% | 17.34% | 56.18% |  |
|  |  |  |  |  |
